# Oclacitinib, a Janus Kinase Inhibitor, Reduces the Frequency of IL-4- and IL-10-, but Not IFN-γ-, Producing Murine CD4^+^ and CD8^+^ T Cells and Counteracts the Induction of Type 1 Regulatory T Cells

**DOI:** 10.3390/molecules26185655

**Published:** 2021-09-17

**Authors:** Agnieszka Jasiecka-Mikołajczyk, Jerzy J. Jaroszewski, Tomasz Maślanka

**Affiliations:** Department of Pharmacology and Toxicology, Faculty of Veterinary Medicine, University of Warmia and Mazury in Olsztyn, Oczapowskiego Street 13, 10-719 Olsztyn, Poland; jerzyj@uwm.edu.pl (J.J.J.); tomasz.maslanka@uwm.edu.pl (T.M.)

**Keywords:** oclacitinib, JAK, CD4^+^ cells, CD8^+^ cells, cytokines

## Abstract

The purpose of the present study was to broaden the knowledge and understanding of the effects of oclacitinib (OCL), a Janus kinase inhibitor, on T cells in the context of both the immune mechanisms underlying anti-inflammatory and anti-allergic properties of the drug and its safety. The results indicate that beneficial effects of OCL in the treatment of skin allergic diseases may be partially mediated by the inhibition of IL-4 production in CD4^+^ and CD8^+^ T cells. To a certain extent, the antiproliferative effect of OCL on CD8^+^ T cells may also contribute to its therapeutic effect. The study found that OCL does not affect the proliferation of CD4^+^ T cells or the number of IFN-γ- and IL-17-producing CD4^+^ and CD8^+^ T cells. Moreover, OCL was found to counteract the induction of type 1 regulatory T (Tr1) cells and to act as a strong inhibitor of IL-10 production in both CD4^+^ and CD8^+^ T cells. Thus, these results indicate that beneficial effects of OCL in the treatment of skin allergic diseases are not mediated through: (a) the abolishment of IFN-γ and IL-17-production in CD4^+^ and CD8^+^ T cells; (b) generation of Tr1 cells; (c) inhibition of CD4^+^ T cell proliferation; (d) induction of IL-10 production in CD4^+^ T cells. The results of this study strongly suggest that, with respect to the evaluated parameters, OCL exerts a suppressive effect on Th2- but not Th1-mediated immunity.

## 1. Introduction

Oclacitinib (OCL) is a novel immunomodulatory agent that has been registered for the treatment of pruritus in allergic dermatitis and atopic dermatitis (AD) in dogs. Although OCL is increasingly used in veterinary medicine, its influence on the immune system is relatively poorly known. Generally, OCL acts as an inhibitor of the function of important pro-inflammatory, pro-allergic and pruritogenic cytokines via inhibition of the Janus kinase (JAK) signal transducer and activator of transcription (STAT) signaling pathway [1]. JAKs constitute a group of 4 enzymes that, by activating STATs, transduce pro-inflammatory and pro-proliferative signals and translate them into an effector function by inducing expression of target genes [2]. In light of the above, the mechanism of OCL action would rely solely on the inhibition of cytokine-mediated signaling via blockade of the JAK-STAT signaling pathway. It is known, however, that the JAK-STAT signaling pathway is also involved in the regulation of cytokine production [3], and that JAK inhibitors may produce an inhibitory or stimulating effect on the production of cytokines [4,5].

AD is one of the most common allergic dermatoses diagnosed in dogs. The pathophysiology of AD is complex. T-helper type 2 (Th2) cytokines (such as IL-4, IL-5, IL-13 and IL-31), but also T-helper type 1(Th1) cytokines (such as IFN-γ and TNF-α) are overproduced in allergic dermatitis in humans and play an important role in the pathogenesis of this disease [6,7,8]. It can be claimed with high probability that dogs suffering from allergic dermatoses, like humans, experience cytokine dysregulation [9,10]. Canine AD is also associated with overproduction of IL-4 [11,12] and IFN-γ [12,13]. Moreover, recent research demonstrated a significant increase in concentrations of circulating IL-17 in dogs with AD [14]. IL-17, produced by CD4^+^ T cells (i.e., Th17 cells), is a well-known proinflammatory cytokine, which enhances the migration of T cells to the skin [15]. Thus, these findings strongly suggest that Th2, but also Th1 and Th17 immune responses contribute to the pathogenesis of canine AD. Although CD4^+^ T cells play a dominant role in the pathogenesis of AD, a few studies have revealed that CD8^+^ T cells are also involved in the development of the disease in dogs [16,17,18].

IL-10 is a crucial anti-inflammatory and immunosuppressive cytokine that plays a suppressive role in AD, and is involved in peripheral T cell tolerance to allergens and autoantigens [19]. Decreased IL-10 expression was associated with AD in humans [20] and dogs [18,21]. IL-10 can be produced by various types of immune cells, including T cells as Th2, Foxp3^+^CD25^+^CD4^+^ regulatory (Treg) cells and type 1 regulatory (Tr1) cells and CD8^+^ cells [22]. Tr1 cells may represent particularly attractive therapeutic targets for AD because they play a pivotal role in restraining human T-cell responses toward environmental allergens and in protecting against allergic diseases [23,24]. It has been shown that some immunosuppressive drugs induced the generation of Tr1 cells from naïve CD4^+^ T cells [25]. Hence, up-regulation of IL-10 production and/or generation of inducible Tr1 cells should translate into the impairment/reduction of the development of immune and inflammatory responses in AD. Taking all of the above into consideration, IL-4, IL-17, IL-10 and IFN-γ as well as Tr1 cells can be considered as the potential targets in the therapeutic strategy of canine AD.

JAK-STAT pathway constitutes a pro-proliferative signaling pathway. It was demonstrated that certain JAK inhibitors exerted antiproliferative effects on T cells [26,27]. Therefore, we hypothesized that OCL can suppress proliferation of CD4^+^ and CD8^+^ T cells.

Taking into consideration the context discussed above, the essential purpose of this study was to determine whether OCL has the potential to inhibit the pathogenesis of allergic dermatitis and AD by: (a) abolition/reduction of IFN-γ, IL-4 and IL-17 production in CD4^+^ (i.e., in Th1, Th2 and Th17) and CD8^+^ (i.e., in Tc cells type 1 (Tc1), type 2 (Tc2) and type 17 (Tc17)) T cells, i.e., important cytokines which may promote the development of these diseases; (b) induction of Tr1 cell generation and IL-10 production in T cells and, i.e., important immunosuppressive cells and cytokine, respectively; (c) inhibiting proliferation of CD4^+^ and CD8^+^ T cells. However, these cells and their cytokines cells have a crucial physiological functions, i.e., they play a essential role in control of microbial infections and cancer development [28]. Thus, the purpose of the present study was to broaden the knowledge and understanding of the effects of OCL on T cells in the aspect of both the immune mechanisms underlying anti-inflammatory and anti-allergic properties of the drug and its safety in the aspect of its possible adverse effect on T cell-mediated immunity.

## 2. Results

### 2.1. OCL Reduces the Frequency of IL-4-, but Not IFN-γ- and IL-17-, Producing CD4^+^ and CD8^+^ T Cells

**Figure 1:**
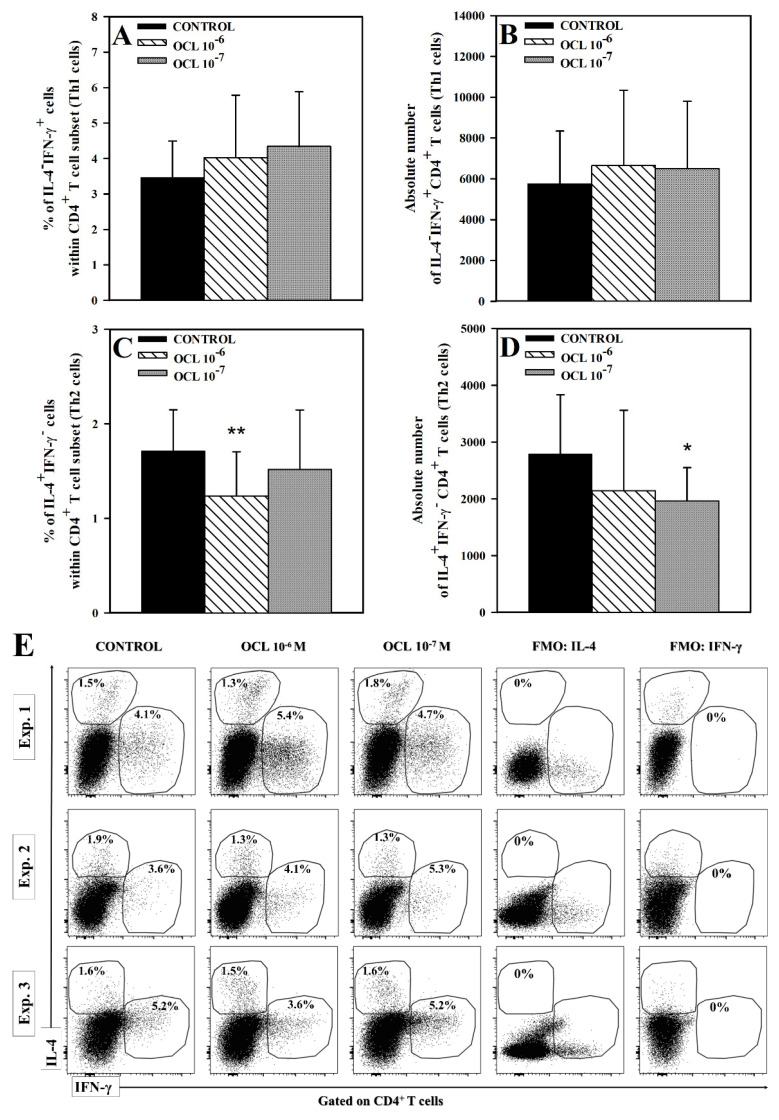
Effect of oclacitinib (OCL) on the number of IFN-γ- and IL-4-producing CD4^+^ T cells. The relative (**A**,**C**) and absolute count (**B**,**D**) of IFN-γ- and IL-4-producing CD4^+^ T cells were determined in cell cultures incubated with or without (control) OCL (10^−6^ M and 10^−7^ M). The relative count is expressed as a percentage of IFN-γ- or IL-4-producing cells within CD4^+^ T cells. The absolute count represents the number of IL-4^−^IFN-γ^+^CD4^+^ and IL-4^+^IFN-γ^−^CD4^+^ T cells per sample well. Cells with such phenotypes should be equated with Th1 and Th2 cells, respectively. Results are expressed as the mean (± S.D.) of three independent experiments with 5 mice per experiment (overall n = 15, * *p* < 0.05, ** *p* < 0.01, control cells vs OCL-treated (10^−6^ or 10^−7^ M) cells (Student’s unpaired *t*-test). Examples of dot plot cytograms show the distribution of IFN-γ- and IL-4-producing and non-producing cells within CD4^+^ T cell subset (**E**). Fluorescence minus one (FMO) controls were applied to confirm the gating strategy used to identify IFN-γ^+^ and IL-4^+^ cells.

**Figure 2:**
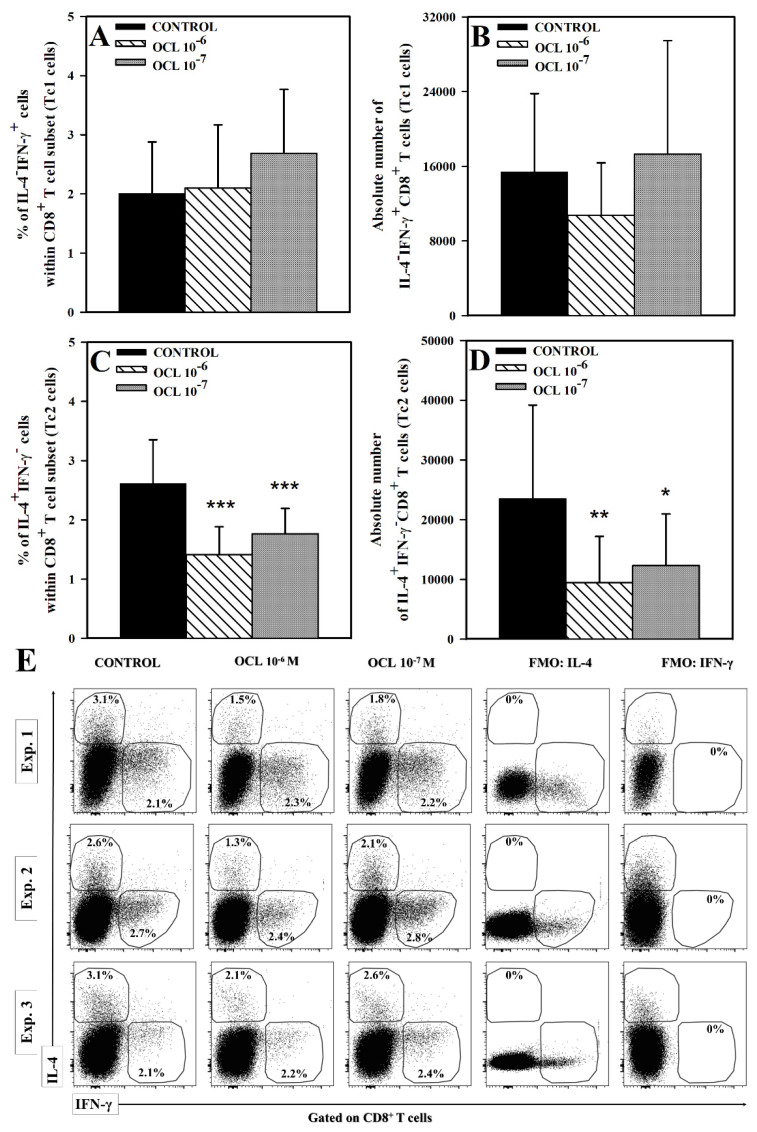
Effect of oclacitinib (OCL) on the number of IFN-γ- and IL-4-producing CD8^+^ T cells. The relative (**A**,**C**) and absolute count (**B**,**D**) of IFN-γ- and IL-4-producing CD8^+^ T cells were determined in cell cultures incubated with or without (control) OCL (10^−6^ M and 10^−7^ M). The relative count is expressed as a percentage of IFN-γ- or IL-4- producing cells within CD8^+^ T cells. The absolute count represents the number of IL-4^−^IFN-γ^+^CD8^+^ and IL-4^+^IFN-γ^−^CD8^+^ T cells per sample well. Cells with such phenotypes should be equated with Tc1 and Tc2 cells, respectively. Results are expressed as the mean (± S.D.) of three independent experiments with 5 mice per experiment (overall n = 15, * *p* < 0.05, ** *p* < 0.01, *** *p* < 0.001, control cells vs OCL-treated (10^−6^ or 10^−7^ M) cells (Student’s unpaired *t*-test)). Examples of dot plot cytograms show the distribution of IFN-γ- and IL-4-producing and non-producing cells within CD8^+^ T cell subset (**E**). Fluorescence minus one (FMO) controls were applied to confirm the gating strategy used to identify IFN-γ^+^ and IL-4^+^ cells.

**Figure 3:**
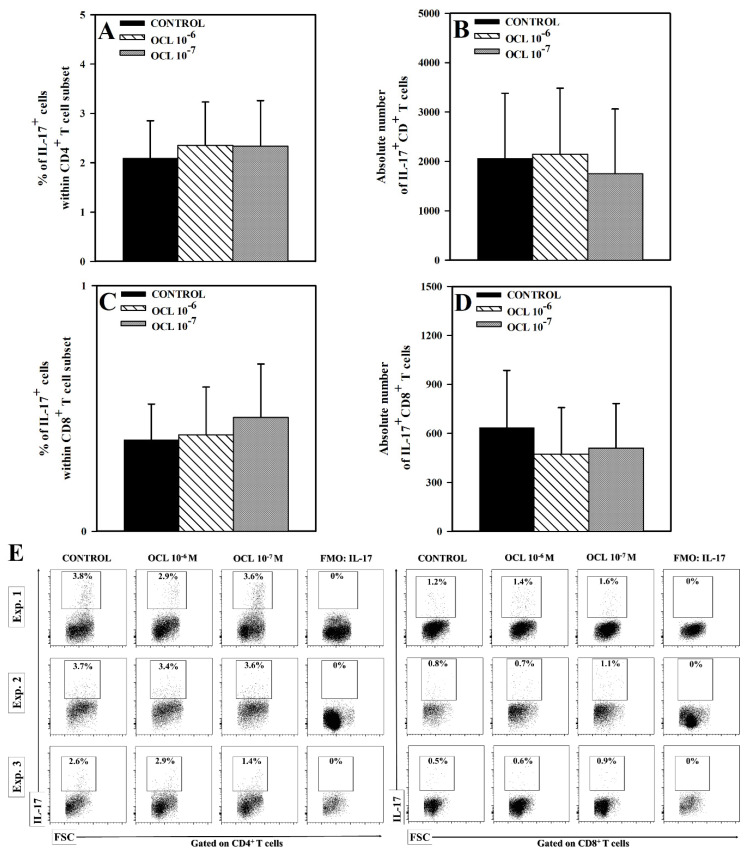
Effect of oclacitinib (OCL) on the number of IL-17-producing CD4^+^ and CD8^+^ T cells. The relative (**A**,**C**) and absolute count (**B**,**D**) of IL-17-producing CD4^+^ and CD8^+^ T cells were determined in cell cultures incubated with or without (control) OCL (10^−6^ M and 10^−7^ M). The relative count is expressed as a percentage of IL-17-producing cells within CD4^+^ and CD8^+^ T cells. The absolute count represents the number of IL-17^+^CD4^+^ and IL-17^+^CD8^+^ T cells per sample well. Cells with such phenotypes should be equated with Th17 and Tc17 cells, respectively. Results are expressed as the mean (±S.D.) of three independent experiments with 5 wells per experiment (overall n = 15). Examples of dot plot cytograms show the distribution of IL-17-producing and non-producing cells within CD4^+^ and CD8^+^ T cell subset (**E**). Fluorescence minus one (FMO) controls were applied to confirm the gating strategy used to identify IL-17^+^ cells.

No effect of OCL (at either concentration) on the percentage and absolute count of IL-4^−^IFN-γ^+^CD4^+^ cells was observed (Figure 1A,B,E). Exposure of cells to OCL 10^−6^ M reduced the percentage of IL-4^+^IFN-γ^−^ cells within CD4^+^ T cell subset (*p* < 0.01), but did not significantly affect the absolute number of these cells, although, a certain trend toward this effect was observed (*p* = 0.164, the mean value of this parameter was 2792 (control) versus 2143 (OCL 10^−6^ M); Figure 1C–E). On the contrary, OCL at the lower concentration did not affect the percentage of IL-4^+^IFN-γ^−^CD4^+^ cells, but reduced the absolute count of these cells (*p* < 0.05; Figure 1C–E). The studies did not reveal a statistically significant effect of OCL on the percentage and absolute count of IL-4^−^IFN-γ^+^CD8^+^ T cells. Exposure of cells to OCL considerably reduced the percentage of IL-4^+^IFN-γ^−^ cells within CD8^+^ T cell subset (*p* < 0.001; Figure 2C) and an absolute count of these cells (*p* < 0.01 (10^−6^ M), *p* < 0.05 (10^−7^ M); Figure 2D). No effect of OCL (at either concentration) on the percentage and absolute count of IL-17-producing CD4^+^ and CD8^+^ T cells was found (Figure 3). The results indicate that OCL did not affect the number of CD4^+^ and CD8^+^ T cells capable of IFN-γ and IL-17 production but abolished IL-4 production in a significant portion of CD4^+^ and CD8^+^ T cells.

### 2.2. OCL Reduces the Percentage and Absolute Counts of IL-10^+^CD4^+^ and IL-10^+^CD8^+^ T Cells

**Figure 4:**
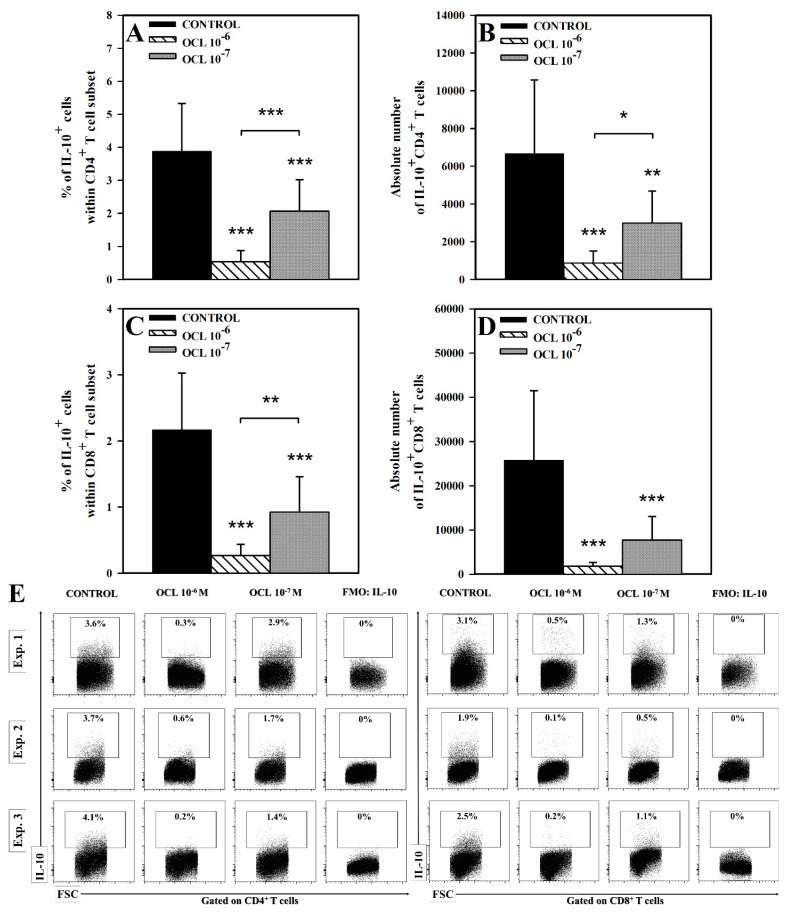
Effect of oclacitinib (OCL) on the number of IL-10-producing CD4^+^ and CD8^+^ T cells. The relative (**A**,**C**) and absolute count (**B**,**D**) of IL-10-producing CD4^+^ and CD8^+^ T cells were determined in cell cultures incubated with or without (control) OCL (10^−6^ M and 10^−7^ M). The relative count is expressed as a percentage of IL-10-producing cells within CD4^+^ and CD8^+^ T cells. The absolute count represents the number of IL-10^+^CD4^+^ and IL-10^+^CD8^+^ T cells per sample well. Results are expressed as the mean (±S.D.) of three independent experiments with 5 mice per experiment (overall n = 15, * *p* < 0.05, ** *p* < 0.01, *** *p* < 0.001, control cells vs OCL-treated (10^−6^ or 10^−7^ M) cells (Student’s unpaired *t*-test), or OCL 10^−6^-treated vs OCL-10^−7^-treated cells (one way ANOVA with Holm-Sidak multiple comparison test)). Examples of dot plot cytograms show the distribution of IL-10-producing and non-producing cells within CD4^+^ and CD8^+^ T cell subset (**E**). Fluorescence minus one (FMO) controls were applied to confirm the gating strategy used to identify IL-10^+^ cells.

The research demonstrated that exposure of cells to OCL in both concentrations caused a significant reduction in the percentage of IL-10^+^ cells within both CD4^+^ and CD8^+^ T cell subsets (*p* < 0.001; Figure 4A,C), as well as in the absolute counts of IL-10^+^CD4^+^ (*p* < 0.001 (10^−6^ M), *p* < 0.01 (10^−7^ M); Figure 4B) and IL-10^+^CD8^+^ (*p* < 0.001; Figure 4D,E) T cells. The OCL 10^−6^ M-induced reduction in the absolute counts of IL-10-producing CD4^+^ and CD8^+^ T cells was extremely profound, because the values of these parameters constituted only 13% and 7% of corresponding control values, respectively. It should be noticed that the percentage (*p* < 0.001) and absolute count (*p* < 0.05) of IL-10-producing CD4^+^ T cells, and the percentage (*p* < 0.01) of IL-10-producing CD8^+^ T cells were significantly lower in the cultures treated with OCL 10^−6^ M compared to those exposed to OCL 10^−7^ M. Thus, it can be stated that, under the condition of this study, OCL 10^−6^ M almost completely abolished IL-10 production in CD4^+^ and CD8^+^ T cells. Moreover, the results obtained indicate that this inhibitory effect on IL-10 synthesis can be dose-dependent.

### 2.3. OCL Reduces the Percentage and Absolute Count of CD49b^+^CD223^+^CD4^+^ T Cells

**Figure 5:**
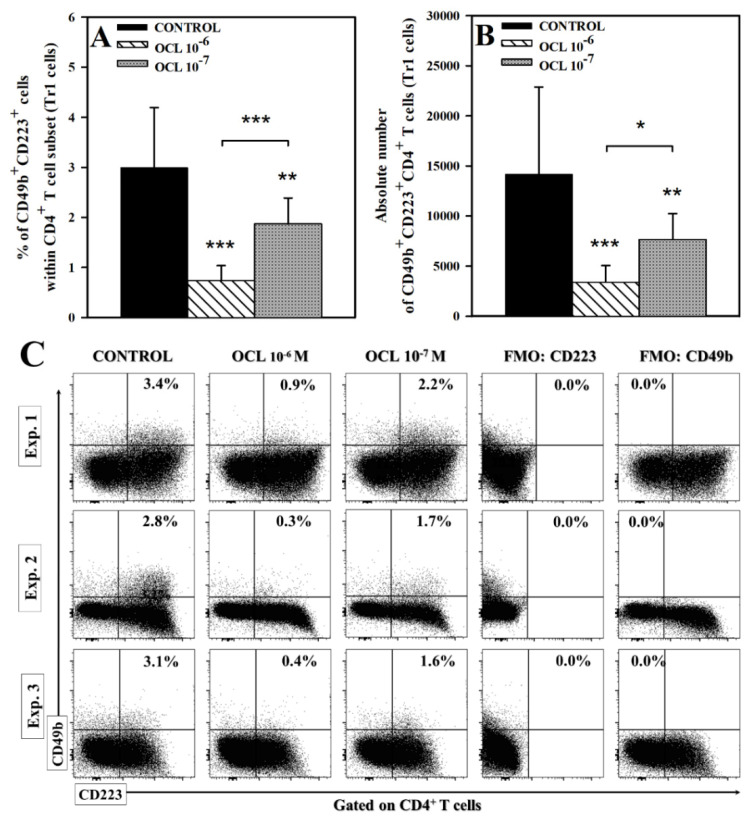
Effect of oclacitinib (OCL) on the number of CD49b^+^CD223^+^CD4^+^ T cells. The relative (**A**) and absolute count (**B**) of CD49b^+^CD223^+^CD4^+^ cells were determined in cell cultures incubated with or without (control) OCL (10^−6^ M and 10^−7^ M). The relative count is expressed as a percentage of CD49b^+^CD223^+^ cells within CD4^+^ T cell subset. The absolute count represents the number of CD49b^+^CD223^+^CD4^+^ T cells per sample well. Cells with such a phenotype should be equated with Tr1 cells. Results are expressed as the mean (±S.D.) of three independent experiments with 5 mice per experiment (overall n = 15, * *p* < 0.05, ** *p* < 0.01, *** *p* < 0.001, control cells vs OCL-treated (10^−6^ or 10^−7^ M) cells (Student’s unpaired *t*-test), or OCL 10^−6^-treated vs OCL-10^−7^-treated cells (one way ANOVA with Holm-Sidak multiple comparison test)). Examples of dot plot cytograms show the distribution of CD49b^+^CD223^+^ cells among CD4^+^ T cell subset (**C**). Fluorescence minus one (FMO) controls were applied to confirm the gating strategy used to identify CD49b^+^CD223^+^ cells.

Treatment with OCL led to a significant reduction in the percentage and the absolute count of CD49b^+^CD223^+^CD4^+^ T cells (*p* < 0.001 (10^−6^ M), *p* < 0.01 (10^−7^ M); Figure 5). It was demonstrated that the values of these parameters were considerably lower in the cultures treated with OCL 10^−6^ M compared to those exposed to OCL 10^−7^ M (*p* < 0.001 and *p* < 0.05, respectively). The drug-induced reduction in the absolute count of CD49b^+^CD223^+^CD4^+^ T cells was very profound, because the values of these parameter in the samples from the cultures treated with OCL 10^−6^ M and 10^−7^ M constituted 25% and 55%, of the corresponding control values, respectively. These results indicate that OCL counteracted the induction of CD49b^+^CD223^+^CD4^+^ T cells and this effect was dose-dependent.

### 2.4. OCL Reduces the Number of Proliferating CD8^+^, but Not CD4^+^, T Cells

**Figure 6:**
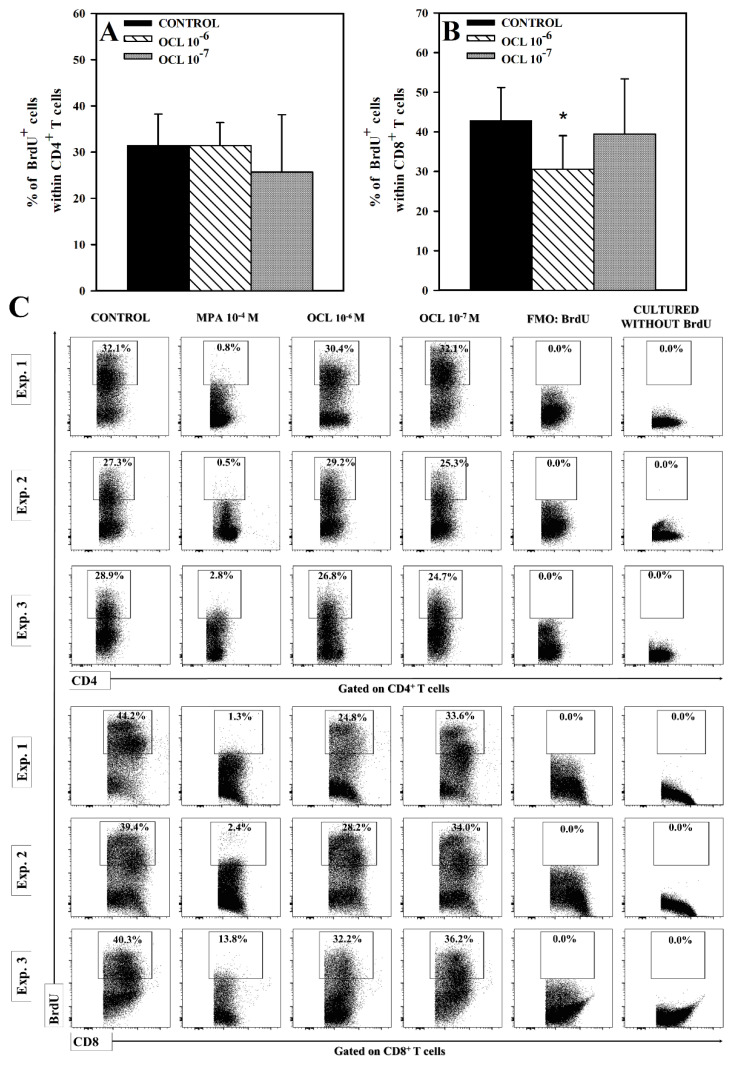
Effect of oclacitinib (OCL) on the proliferation of CD4^+^ (**A**) and CD8^+^ (**B**) T cells. The relative count of 5-bromo-2’-deoxyuridine(BrdU)-incorporating cells among CD4^+^ and CD8^+^ T cells was determined in cell cultures incubated with or without (control) OCL (10^−^^6^ M and 10^−^^7^ M). Results are the mean (± S.D.) of three independent experiments with 5 mice per experiment (overall n = 15, * *p* < 0.001, control cells vs OCL-treated (10^−6^ or 10^−7^ M) cells (Student’s unpaired *t*-test)). Examples of dot plot cytograms show the distribution of BrdU-positive and -negative cells within CD4^+^ and CD8^+^ T cell subsets (**C**). Fluorescence minus one (FMO) controls as well as cultured cells without BrdU were used to confirm the gating strategy used to identify BrdU^+^ cells. Cells treated with mycophenolic acid (MPA, an active metabolite of the prodrug mycophenolate mofetil), i.e., the agent with the proved antiproliferative activity to T cells, constituted an additional negative as well as a reference control.

No effect of OCL on the percentage of 5-bromo-2’deoxyuridine(BrdU)^+^ cells within CD4^+^ T cell was observed (Figure 6A,C). However, the studies demonstrated that OCL at 10^−6^ M concentration, but not at 10^−7^ M, reduced the percentage of BrdU-incorporating cells within CD8^+^ T cell subset (*p* < 0.001; Figure 6B). The results indicate that OCL diminished the proliferation of CD8^+^, but not CD4^+^, T cells.

## 3. Discussion

This work is a continuation of our research [29] on an influence of OCL on T cells, which was conducted on canine peripheral blood mononuclear cells (PBMCs), i.e., on cells of the target species for which the drug (APOQUEL) has been specifically approved. In order to achieve research objectives formulated in this paper, the present study by necessity was conducted on mouse lymphocytes because anti-canine monoclonal antibodies for flow cytometric detection of Tr1 cells and intracellular cytokine production as well as to obtain effective stimulation of cells (to induce cell proliferation and cytokine synthesis) are still not available for dogs.

The present study did not demonstrate the effect of OCL on the percentage and absolute count of IFN-γ-producing CD4^+^ and CD8^+^ T cells and IL-17-producing CD4^+^ and CD8^+^ T cells; cells with such a phenotype should be equated with Th1, Tc1, Th17 and Tc17 cells, respectively. Thus, these results indicate that OCL did not abolish or induce the capacity of these cells to produce IFN-γ and IL-17. It can therefore be stated that the results obtained in this experiment indicate that the therapeutic effect of OCL is not associated with the abolishment of IFN-γ and IL-17-production, i.e., two important pro-inflammatory cytokines, in CD4^+^ and CD8^+^ T cells. Some reports indicate that STAT4 [30,31] and STAT5b [32] activation is involved in the regulating IFN-γ production in CD4^+^ T cells. In turn, STAT3 can increase the production of IL-17 in these cells while STAT5 opposes this process [33]. However, the results obtained strongly suggest that the pharmacological manipulation of JAK-STAT activity, by means of OCL, does not affect this signaling cascade with respect to its involvement in the regulation of IFN-γ and IL-17 production by CD4^+^ and CD8^+^ T cells.

Exposure of cells to OCL led to a decrease in the percentage (10^−6^ M) and absolute count (10^−7^ M) of IL-4-producing CD4^+^ T cells, i.e., Th2 cells, and the mean value of these parameters constituted about 72% and 70% of the control values. What is more, OCL at both concentrations significantly reduced the percentage and absolute count of IL-4-producing CD8^+^ T cells, i.e., Tc2 cells. The mean percentage and absolute count of these cells in samples obtained from cultures exposed to OCL 10^−6^ M constituted about 54% and 40% of the control values. With a certain measure of simplification, cells of the phenotypes IL-4^+^CD4^+^ and IL-4^+^CD8^+^ can be equated with Th2 and Tc2 cells, respectively. Thus, the reductive effect of OCL on the number of IL-4 producing cells was definitely much more pronounced for Tc2 than for Th2 T cells. Considering the pathogenic role of IL-4 in AD (and in other allergic dermatitis), IL-4 signaling is regarded as a promising potential therapeutic target for the treatment of AD [34]. Although CD4^+^ T cells, including naïve and Th2 cells, are regarded as the crucial source of IL-4 during Th2 responses, CD8^+^ T cells can also contribute to the overproduction of IL-4 in AD. The researches of recent years indicate that AD in humans is accompanied by the recruitment of not only Th2, but also Tc2, cells into the skin [35]. Moreover, it was demonstrated that CD8^+^ T cells are also infiltrated the skin of dogs with AD [16,17]. These results of present studies indicate that OCL can abolish IL-4 production in a significant portion of Th2 and Tc2 cells, respectively. Taking all of the above into consideration, it can be concluded that the beneficial effects of OCL in the treatment of skin allergic diseases may be partially mediated by the inhibition of IL-4 production in Th2 and Tc2 cells. This conclusion is in agreement with the previous studies which demonstrated that generation of IL-4-secreting Th2 and Tc2 cells in vitro is critically dependent on STAT6 signaling [36].

Surprisingly, the present study showed that the percentage and absolute count of IL-10-producing CD4^+^ and CD8^+^ T cells were extremely reduced in the cultures treated with both concentrations of OCL. The percentages of IL-10-producing CD4^+^ and CD8^+^ T cells and absolute counts IL-10-producing CD4^+^ and CD8^+^ T cells in samples from cultures exposed to OCL 10^−6^ M constituted only about 13%, 12%, 13% and 7% of the control values, respectively. These results justify the conclusion that OCL at the higher concentration almost completely abolished IL-10-production in CD4^+^ and CD8^+^ T cells. It is reasonable to believe that this effect may be associated with inhibitory effect of OCL on STAT1 and STAT3 signaling cascade because activation of these STATs have been involved in the induction of IL-10 production in CD4^+^ T cells, including Th [27,37] and Treg [38] cells. Similarly, the mean percentage and absolute count of CD49b^+^CD223^+^CD4^+^ T cells, i.e., Tr1 cells, in samples obtained from cultures exposed to OCL in the higher concentration constituted only about 24% of the control values. Tr1 cells were not detectable in unstimulated cultures. This was not unexpected since Tr1 cells represent an inducible regulatory cells which are generated from naïve CD4^+^ T cells under conditions involving T cell activation [39], as was the case in the present studies. In our previous research it was found that OCL inhibited activation of CD4^+^ T cells [29]. What is more, the findings of certain studies indicate that generation of Tr1 cells can depend on STAT3 activation [40,41]. In light of the above, it should be concluded that OCL prevented the generation of Tr1 cells rather than depleted these cells. The regulatory activity of Tr1 cells (i.e., prevention of excessive inflammatory responses and maintaining immune tolerance) is largely mediated by their production of IL-10, which dampens the function of both antigen-presenting cells and antigen-specific effector T cells [24]. Thus, Tr1 cells are important producers of IL-10 within the CD4^+^ T cell subset, especially under activation conditions. In the light of these results it can be said that the findings concerning the influence of OCL on Tr1 cells and IL-10 production are not only closely related in substance but also they are mutually consistent. In conclusion, the induction of Tr1 cells generation and IL-10 production in T cells do not constitute—as was hypothesized—the modes of action that may be involved in the therapeutic effect of OCL. What is more, paradoxically, the drug with undisputed anti-allergic properties counteracts the generation Tr1 cells and production of IL-10, i.e., important immunosuppressive cells and cytokine playing essential roles in immune tolerance to allergens.

The results indicate that OCL does not exert an antiproliferative effect on CD4^+^ T cells, as could be expected from the JAK inhibitor. Thus, the research hypothesis that an additional mechanism involved in producing anti-inflammatory and anti-allergic properties of OCL might have an inhibitory effect on the proliferation of CD4^+^ T cells (i.e., cells playing a dominant role in the pathogenesis of allergic diseases including AD) was verified negatively. Interestingly, the present study demonstrated that OCL diminished the proliferation of CD8^+^ T cells, although this effect occurred only at the higher concentration and was relatively moderate in magnitude (especially when compared to mycophenolic acid, an immunosuppressant agent with antiproliferative activity against T cells). Although CD4^+^ T cells play a central role in the pathogenesis of AD, a few studies have demonstrated that CD8^+^ T cells are also involved in the development of the disease in dogs [16,17,18]. Therefore, it could be suggested that the antiproliferative effect on CD8^+^ T cells may, to a certain extent, contribute to the anti-allergic efficacy of OCL. 

The results not only deepen our knowledge of mechanisms and immune effects underlying the therapeutic efficacy of OCL, but also shed light on drug safety in terms of its possible adverse effect on T cells (in terms of the evaluated parameters). In general, Th1 cells primarily enhance the cell-mediated immune response to intracellular pathogens through the synthesis of pro-inflammatory Th1 cytokines, especially IFN-γ. Similarly, CD8^+^ T cells, especially Tc1 cells, play a critical role in the immunity against intracellular infections and tumors, and IFN-γ is the main cytokine associated with these protective responses [42]. Although Tc17 cells are known to contribute to the mediation of pathologic inflammation and autoimmune processes, they are also involved in protective immune responses against fungi, viruses and bacteria [43]. The results strongly suggests that OCL does not affect: (a) Th1-mediated immune response with respect to evaluated parameters, i.e., proliferation of CD4^+^ T cells and the number of Th1 cells capable of IFN-γ production; (b) Tc1- and Tc17-mediated immune responses with respect to the number of Tc1 and Tc17 cells capable of IFN-γ and IL-17 production, respectively, but may have the potential to impair these responses via an inhibitory effect on proliferation of CD8^+^ T cells. However, it does not preclude a negative effect of the drug on Th1 cells. In our previous studies conducted on non-stimulated canine PBMCs, we found that OCL exerted a proapoptotic effect on CD4^+^ and CD8^+^ T cells and caused depletion of these cells [29].

Th2-mediated immunity protects against extracellular pathogens and helminths [44]. This study revealed that OCL reduced the number of CD4^+^ T cells capable of IL-4 and IL-10 production, i.e., Th2 hallmark cytokines. Thus, in terms of the estimated parameters, the results obtained strongly suggest that OCL may exert the suppressive effect on Th2-mediated immunity. 

To the best of our knowledge, the available literature lacks any reports on the effect of OCL on proliferation of CD4^+^ and CD8^+^ T cells and on production cytokines by these subsets (i.e., on the number of cytokine-producing CD4^+^ or CD8^+^ T cells). There is a research which demonstrated that exposure of mitogen stimulated canine PBMCs on OCL did not result in any change in the level of IFN-γ and IL-10 in culture supernatants, although the drug decreased proliferation of these cells [45]. However, the cited studies do not provide adequate data for comparison with our results because PBMCs, beside T cells, include B cells, NK cells, dendritic cells and monocytes, that is the cells which may also produce IFN-γ and IL-10 and other cytokines. Whereas in the present study the effect of OCL on cytokine production and cell proliferation was evaluated with respect to the number of cytokine-producing and proliferating, respectively, CD4^+^ and CD8^+^ T cells. Hammitzsch et al. [5] demonstrated that tofacitinib (i.e., JAK1/JAK3 inhibitor), baricitinib and ruxolitinib (i.e., JAK1/JAK2 inhibitors) decreased IL-17 production by CD4^+^ T cells, whereas in present study OCL did not affect the number of IL-17-producing CD4^+^ T cells. Ghoreschi et al. [46] demonstrated that exposure of murine CD4^+^ T cells on tofacitinib did not affect proliferation of these cells but decreased their IFN-γ production; these results are, respectively, consistent and inconsistent with ours. Any deeper and broader comparison or discussion of our results relative to results from similar studies with the use of other JAK inhibitors would exceed the scope of this paper. However, all the evidence suggests that although other JAK inhibitors inhibit the same type of JAK kinase, they may exert different effects on the production of a specific cytokine. Thus, it seems clear that the direction of the effect of JAK inhibitors on cytokine production may not be determined solely by the blockade of a specific JAK but can depend on other factors as well. It should be noted that the role of JAK-STAT pathway in cytokine signal transduction is established, while its involvement in the regulation of cytokine production does not seem to be well studied and understood. It should be also taken into consideration that certain JAK inhibitors may have a more complex mechanism of action, i.e., they may affect cytokine production through other mechanisms than the inhibition of JAK-STAT signaling.

## 4. Materials and Methods

### 4.1. Animals

The experiments were carried out on female 6-week-old Balb/c mice. Mice were bred and maintained under standard laboratory conditions (12/12 h light cycle, controlled temperature (21 ± 2 °C) and humidity (55 ± 5%)) with ad libitum access to food and water, in the Animal Facility of the Faculty of Veterinary Medicine, University of Warmia and Mazury in Olsztyn. Mice were euthanized by asphyxiation with CO_2_. Law in Poland (Act of 15 January 2015 on the Protection of Animals Used for Scientific or Educational Purposes) does not require a permit from an ethics commission to conduct experiments in which samples for research are obtained post mortem from animals not submitted to any procedure while alive.

### 4.2. Isolation of Lymphocytes

#### 4.2.1. Spleen

Spleens were removed and subjected to homogenization in a Dounce tissue grinder. The resulting cell suspensions were filtered through 70 µm cell strainer (BD Biosciences, San Jose, NJ, USA) and washed (300× *g* for 5 min at 5 °C; the same parameters were used for all cell-washing procedures) with complete medium (CM, RPMI-1640 + 10% heat-inactivated fetal bovine serum (FBS) + 10% HEPES buffer + 10 mM non-essential amino-acid + 10% mM sodium pyruvate + 10 U/mL penicillin/streptomycin; all from Sigma Aldrich, Schnelldorf, Germany). Isolated cells were re-suspended in a 40% Percoll solution (100% Percoll solution = 90% of Percoll + 10% of 10× Hanks’ Balanced Salt Solution; both from Sigma-Aldrich; dilutions of Percoll solution were prepared using CM), and then they were layered over a 60% Percoll solution and subjected to gradient centrifugation (400× *g* for 20 min at 20 °C). Mononuclear cells were removed from the interface layer, washed and then re-suspended in CM.

#### 4.2.2. Lungs

The lavaged lungs were removed and subjected to homogenization in a Dounce tissue grinder and washed in CM. Because the cell yield from lung samples of a single mouse is relatively poor, whole lungs obtained from two animals were pooled into one sample. Lung lymphocytes were isolated by enzymatic digest with 50 U/mL (25 mL per sample) collagenase type IV (Sigma-Aldrich, MO, USA) in a 50-mL glass flask for 90 min at 37 °C. Then the supernatant was removed and filtered through 70 µm cell strainer (BD Biosciences, San Jose, NJ, USA) and the cells were washed in CM. Isolated cells were re-suspended in a 40% Percoll solution, and then they were layered over a 60% Percoll solution and subjected to gradient centrifugation (400× *g* for 20 min at 20 °C). Mononuclear cells were removed from the interface layer, washed, and re-suspended in CM.

### 4.3. In-Vitro Stimulation and Culture Conditions

In order to evaluate the effect of OCL on the number of IL-17-producing T cells, lung lymphocytes were used because our laboratory experience indicates that T cells derived from the lungs are better producers of this cytokine compared to splenocytes. For the remaining assays, splenocytes were applied. Spleen and lung lymphocytes were adjusted to 3 × 10^6^/mL and 1 × 10^6^ cells/mL in CM, respectively, and seeded in 24-well plates in 1 mL aliquots. To eliminate the influence of individual differences between animals, the same cells were used as both control and treated cells. Cells were activated with plate-coated anti-CD3 (Purified NA/LE hamster anti-mouse CD3e, 1 μg/mL, clone 145-2C11) and soluble anti-CD28 (Purified NA/LE hamster anti-mouse CD28, 1 μg/mL, clone 37.51) in the presence of IL-2 (Recombinant mouse IL-2, 20 ng/mL; all reagents from BD Biosciences). In each experiment, cells were exposed to OCL (analytical standard; >98%; Cayman Chemical, MI, USA) in concentration reflecting its plasma levels achieved in vivo at a typical dose (10^−6^ M) [47] and in a ten-fold lower concentration (10^−7^ M). OCL was dissolved in DMSO; therefore, the same amount of DMSO was added to control wells. Cells were incubated for 72 h, followed by re-stimulation with phorbol-12-myristate-13-acetate (PMA; 50 ng/mL) and ionomycin (1 µg/mL; both from Sigma-Aldrich, MO, USA) for the last 5 h. Brefeldin A (Protein transport inhibitor, 1 µL/mL; BD Biosciences, San Jose, NJ, USA) was added for final 4 h of culture to inhibit cytokine release by cells. The plates were incubated at 37 °C in an atmosphere of a humidified incubator with 5% CO_2_ and 95% air. Cell proliferation was evaluated in the presence of BrdU (APC BrdU Flow Kit, BD Biosciences, San Jose, NJ, USA) at a final concentration of 100 µM in cell culture medium during the last 12 h. Each experiment included 5 wells of cells obtained from individual mice (with the exception of wells for evaluation of IL-17 producing cells; these wells contained lung cells pooled from 2 mice) for each condition tested. All experiments were repeated independently three times (overall n = 15).

### 4.4. Flow Cytometry

#### 4.4.1. Extracellular Staining

Cells prepared as described above were stained for surface antigens with fluorochrome conjugated monoclonal antibodies (mAb) specific to mouse CD4, CD8a and CD49b (all from BD Biosciences, San Jose, NJ, USA), as demonstrated in Table 1. After 30 min incubation (on ice and in the dark), the cells were washed in 2 mL of FACS buffer (FB, 1× Dulbecco’s PBS (Sigma-Aldrich) devoid of Ca^2+^ and Mg^2+^ with 2% (*v/v*) heat-inactivated FBS). The staining combinations, properties of antibodies used in the experiments and evaluated parameters are summarized in Table 1.

#### 4.4.2. Intracellular Staining for Determination of IL-4-, IL-10-, IL-17- and IFN-γ-Producing Cells and Tr1 Cells

Cells stained for surface markers (i.e., for CD4, CD8 and CD49b) as described above were fixed and permeabilized using Cytofix/Cytoperm solution and Perm/Wash buffer (both from BD Biosciences, San Jose, NJ, USA) according to the manufacturer’s protocol. Subsequently, the samples were stained with mAb specific to IL-4, IL-10, IL-17, IFN-γ (in order to determine cytokine production) and CD223 (in order to identify Tr1 cells). After 1 h of incubation (at room temperature in the dark) the cells were washed twice with 2 mL of FB and analyzed by flow cytometry.

#### 4.4.3. Intracellular Staining for Determination of Proliferating Cells 

Cells were stained for surface markers (i.e., for CD4 and CD8) as described above and thereafter stained for incorporated BrdU according to the manufacturer’s procedure (APC BrdU Flow Kit, BD Biosciences, San Jose, NJ, USA).

### 4.5. FACS Acquisition and Data Analysis

**Figure 7:**
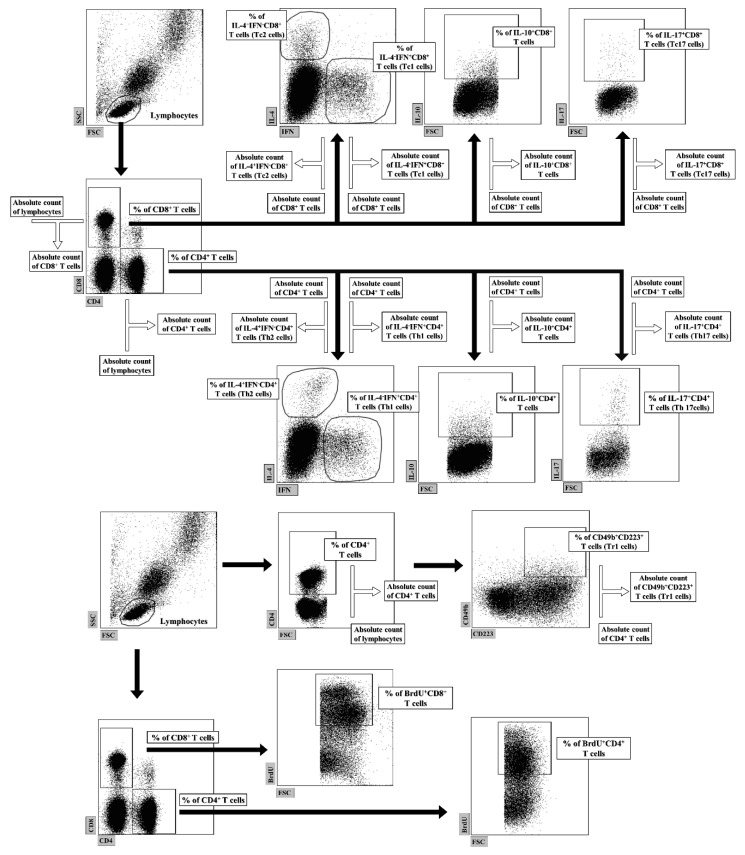
Gating strategy for flow cytometric data analysis and calculation of the absolute cell counts of lymphocyte subsets. Lymphocytes were identified based on forward and side scatter (FSC/SSC) properties, and then gated for expression of CD4 or CD8 surface receptors. Subsequently, IL-4-, IL-10-, IL-17- and IFN-γ-producing cells as well as CD49b^+^CD223^+^-expressing and 5-bromo-2’-deoxyuridine(BrdU)-incorporating cells were identified within particular T cell subsets. Absolute cell counts of lymphocyte subsets (i.e., number of cells from particular subpopulations per sample well) were calculated using the dual platform method, as shown above.

Flow cytometry analysis was performed using a FACSCelesta cytometer (BD Biosciences, San Jose, NJ, USA). The data were acquired by FACSDiva version 9.0 software (BD Biosciences, San Jose, NJ, USA) and analyzed by FlowJo software (Tree Star Inc., Stanford, CA, USA). Absolute cell counts (i.e., number of cells from a particular subpopulation per tissue sample) was calculated using the dual platform method, i.e., the absolute cell count was determined by calculating the data obtained from a cell counting chamber by the percentage of particular cell subsets, as illustrated in Figure 7.

### 4.6. Statistical Analysis

All data are presented as a mean (±S.D.). For comparison of two groups (OCL-treated (at either concentration)) vs. untreated cells, Student’s unpaired *t*-test was used. Additional comparison between all groups was performed (one way ANOVA with Holm-Sidak multiple comparison test) if the drug at both concentrations was shown to affect the parameter. Differences were deemed significant when the *p* values were <0.05. The data were graphed with Sigmaplot software (version 12, Systat Software, Inc., CA, USA).

## 5. Conclusions

The beneficial effects of OCL in the treatment of skin allergic diseases may be partially mediated by the inhibition of IL-4 production in Th2 and Tc2 cells. It should be taken into consideration that the antiproliferative effect on CD8^+^ T cells may also contribute, to a certain extent, to the therapeutic effect of OCL. The present study found that OCL does not affect: (a) the proliferation of CD4^+^ T cells; (b) the number of IFN-γ-producing Th1 and Tc1 cells; (c) the number of IL-17-producing Th17 and Tc17 cells. Moreover, paradoxically, OCL was found to counteract the induction of Tr1 cells and act as a strong inhibitor of IL-10 production in both CD4^+^ and CD8^+^ T cells. Thus, these results indicate that beneficial effects of OCL in the treatment of skin allergic diseases is not mediated through: (a) the abolishment of IFN-γ and IL-17-production in CD4^+^ and CD8^+^ T cells; (b) generation of Tr1 cells; (c) inhibition of CD4^+^ T cell proliferation; (d) induction of IL-10 production in CD4^+^ T cells. The lack of any effect on IFN-γ production and the downregulating effect on IL-4 and IL-10 production in CD4^+^ T cells strongly suggest that—with respect to these parameters—OCL exerts a suppressive effect on Th2-, but not Th1-, mediated immunity.

## Figures and Tables

**Table 1:** The staining combinations, characteristics of monoclonal antibodies and evaluated parameters.

Marker	Fluorochrome	Clone	Isotype	Evaluated Parameters
CD4	FITC	H129.19	IgG2a, κ	The percentage and absolute count of: ➢ IL-4^−^IFN-γ^+^CD4^+^ T cells (Th1 cells) ➢ IL-4^+^IFN-γ^−^CD4^+^ T cells (Th2 cells) ➢ IL-4^−^IFN-γ^+^CD8^+^ T cells (Tc1 cells) ➢ IL-4^+^IFN-γ^−^CD8^+^ T cells (Tc2 cells) ➢ IL-10^+^CD4^+^ T cells ➢ IL-10^+^CD8^+^ T cells
CD8a	APC-Cy7	53-6.7	IgG2a, κ	
IL-4	PE-CF594	11B11	IgG1	
IL-10	APC	JES5-16E3	IgG2b	
IFN-γ	AF700	XMG1.2	IgG1,κ	
CD4	FITC	H129.19	IgG2a, κ	The percentage and absolute count of: ➢ IL-17^+^CD4^+^ T cells (Th17 cells) ➢ IL-17^+^CD8^+^ T cells (Tc17 cells)
CD8a	APC-Cy7	53-6.7	IgG2a, κ	
IL-17	PerCP-Cy5.5	TC11-18H10	IgG1, λ	
CD4	FITC	H129.19	IgG2a, κ	The percentage and absolute count of: ➢ CD49b^+^CD223^+^CD4^+^ T cells (Tr1 cells)
CD49b	PE-CF594	DX5	IgM, κ	
CD223	BB700	C9B7W	IgG1, κ	
CD4	FITC	H129.19	IgG2a, κ	The percentage of: ➢ BrdU-incorporating CD4^+^ and CD8^+^ T cells (i.e., proliferating cells)
CD8a	APC-Cy7	53-6.7	IgG2a, κ	
BrdU	APC	BU1/75	IgG2a	

## Data Availability

The data presented in this study are available on reasonable request from the corresponding author.

## Sample Availability

Samples of the compound are not available from the authors.
